# The critical role of Sirt1 in ischemic stroke

**DOI:** 10.3389/fphar.2025.1425560

**Published:** 2025-03-14

**Authors:** Ziyi Jia, Ke Xu, Ruobing Li, Siyu Yang, Long Chen, Qianwen Zhang, Shulin Li, Xiaowei Sun

**Affiliations:** ^1^ The First Clinical Medical College, Heilongjiang University of Chinese Medicine, Harbin, China; ^2^ The Second Clinical Medical College, Heilongjiang University of Chinese Medicine, Harbin, China; ^3^ The Fourth Clinical Medical College, Heilongjiang University of Chinese Medicine, Harbin, China; ^4^ The First Affiliated Hospital, Heilongjiang University of Chinese Medicine, Harbin, China

**Keywords:** ischemic stroke, SIRT1, inflammation, oxidative stress, autophagy, mitochondrial dysfunction, pan-apoptosis

## Abstract

Ischemic stroke, the most prevalent form of stroke, is responsible for the highest disability rates globally and ranks as the primary cause of mortality worldwide. Sirt1, extensively investigated in neurodegenerative disorders, is the most well-known and earliest member of the sirtuins family. However, its mechanism of action during ischemic stroke remains ambiguous. The literature examination revealed the intricate involvement of Sirt1 in regulating both physiological and pathological mechanisms during ischemic stroke. Sirt1 demonstrates deacetylation effects on PGC-1α, HMGB1, FOXOs, and p53. It hinders the activation of NLRP3 inflammasome and NF-κB while also engaging with AMPK. It regulates inflammatory response, oxidative stress, mitochondrial dysfunction, autophagy, pro-death, and necrotic apoptosis. Therefore, the potential of Sirt1 as a therapeutic target for the management of ischemic stroke is promising.

## 1 Introduction

Stroke ranks second among the leading causes of global mortality and is identified as the primary contributor to worldwide disability (Saini et al., 2021), causing at least 6 million deaths and affecting 13 million people each year, The predominant type of stroke is ischemic stroke, accounting for over 85% of cases. Its incidence has increased in the past few years (Ma et al., 2023). The etiology of ischemic stroke is multifactorial, with three primary contributors: approximately 50% of cases are attributed to the presence and rupture of atherosclerotic plaques in cerebrovascular arteries, around 20% are associated with cardiogenic cerebral infarction, and about 25% result from lacunar infarction caused by lesions in small blood vessels (Zhao et al., 2022). Additionally, the remaining 5% can be attributed to other specific causes such as vasculitis and dissection of extracranial arteries (Warlow et al., 2003). The main underlying factor is the disruption of cerebral blood flow due to multiple factors, resulting in brain tissue ischemia and hypoxic necrosis along with subsequent neurological impairments. The sudden interruption of blood supply to the brain leads to cellular death within the ischemic core, subsequently resulting in cerebral edema and compromise of the blood-brain barrier (Yemisci et al., 2015). The release of necrotic cells induces apoptosis and inflammatory cytokines, which leads to the death of half of the cells near the infarction and aggravates brain damage (Ren and Yang, 2018), releasing a cascade of pathophysiological processes, including the onset of oxidative stress, autophagy, and neuroinflammation. Ultimately, it leads to neuronal death and dysfunction. The primary therapeutic approach for ischemic brain injury is thrombolytic therapy (Stoll et al., 2008), which aims to restore brain perfusion in time; however, reperfusion may facilitate secondary cell death and exacerbate brain injury, leading to the occurrence of cerebral ischemia/reperfusion injury (Datta et al., 2020). Hence, it is imperative to explore novel options that can extend the time frame and enhance the outlook for individuals suffering from ischemic stroke.

Silent information regulators (Sirtuins) are class III histone deacetylases. The mammalian sirtuins family can be divided into seven types: The Sirt1-Sirt7 proteins exhibit distinct enzymatic activities, physiological functions, and subcellular localization (Batiha et al., 2023). The terminal region expansion of Sirt1is the most significant among all sirtuins. The Sirt1 protein, consisting of 747 amino acids, is composed of a highly conserved catalytic core spanning residues 244 to 512, a COOH terminal region encompassing residues 1 to 180, and an NH2 terminal region spanning residues 513 to 747 (Yang et al., 2022). Therefore, the first identified sirtuin, Sirt1, stands out as the most extensively characterized among the seven sirtuins. A wide range of research has indicated that Sirt1 exhibits extensive expression across diverse human tissues and organs, including brain (Cao et al., 2018), liver (Al-Bahrani et al., 2015), heart (Tanno et al., 2010), skeletal muscle (Goh et al., 2014), pancreas (Tavano et al., 2015) and adipose tissue (Jorge et al., 2017). It is worth mentioning that Sirt1 is present in crucial metabolic hubs within the brain, such as the hypothalamic ramiform nucleus, ventromedial hypothalamic nucleus, dorsal-medial hypothalamic nucleus, paraventricular hypothalamic nucleus, Nucleus tractus solitarius. and other regions associated withneurodegenerative disorders. These include but are not limited to the prefrontal cortex, hippocampus, and basal ganglia. The anatomical studies conducted by Ram et al. in mice demonstrated that the expression of Sirt1 mRNA is predominantly observed in neurons (Ramadori et al., 2008) and that Sirt1 exhibits high expression levels in the spinal cord and dorsal root ganglia, with specificity towards neurons (Yan et al., 2017), so that Sirt1 regulates neuronal differentiation (Watroba and Szukiewicz, 2022), affecting their structure at multiple levels. The expression of inducible nitric oxide synthase (iNOS) is inhibited by Sirt1, thereby reducing neuronal damage. In addition, neural stem cells and neural progenitor cells also contain Sirt1 (Herskovits and Guarente, 2014), astrocytes, microglia (Thompson et al., 2013), and glial cells in the human brain. Under different circumstances, activation and silencing of Sirt1 may lead to neurotrophic changes and cell differentiation depending on the distribution of Sirt1 in different brain regions. Increase neurogenesis or reduce inflammatory states. Previous studies have shown that Sirt1 deacetylates histone H4, histone H3, and histone H1 (Jiao and Gong, 2020). In addition to histones, Sirt1 possesses the ability to facilitate the deacetylation process of numerous non-histone substrates. These include nuclear factor-κb (NF-κB), peroxisome proliferator-activated receptor-γ coactivator-1α (PGC-1α), class O forkhead transcription factors (FOXOs), as well as hypoxia-inducible factor 1a (HIF-1α) (Fujita and Yamashita, 2018). In total, more than 70 crucial proteins undergo this catalytic transformation. By means of these interactions, Sirt1 plays a crucial role in the control of numerous cellular functions and physiological processes, including cell cycle control, energy consumption, oxidative stress (Rajendran et al., 2011), inflammation, apoptosis, mitochondrial biogenesis (Zhao Q et al., 2020), autophagy (Salminen and Fitzgerald, 2009), and aging. It has been reported that patients with neurodegenerative diseases have a more significant reduction in serum Sirt1 protein levels than normal aging individuals (Li et al., 2023). In summary, the participation of Sirt1 in the neuropathogenesis of ischemic stroke suggests its potential as a therapeutic target for managing this condition.

## 2 The mechanism of Sirt1 in ischemic stroke

### 2.1 Sirt1 and inflammation

**FIGURE 1:**
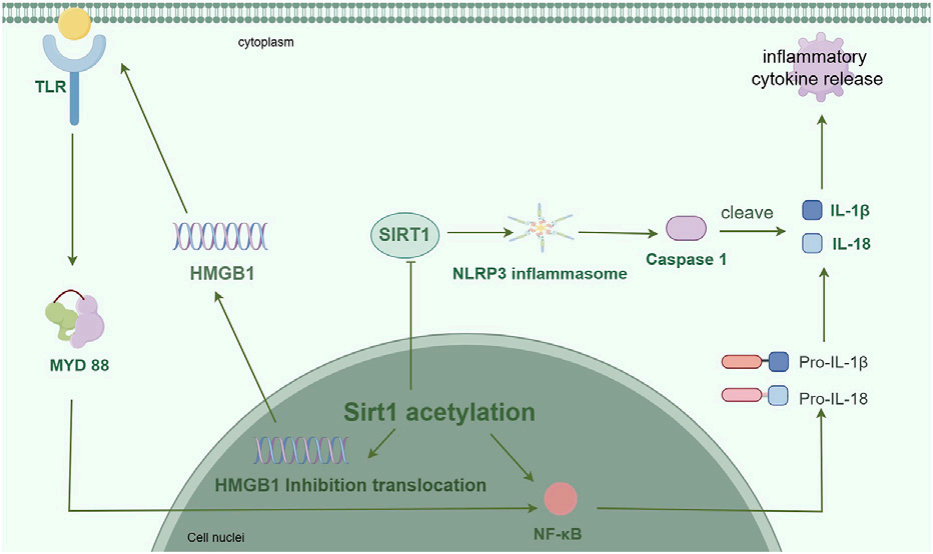
The expression of inflammatory factors is suppressed by Sirt1, which actively engages in the regulation of inflammation through the formation of signaling pathways involving NF-κB, HMGB1, and NLRP3. (By Figdraw).

It is widely acknowledged that inflammation plays a crucial role throughout the entire progression of ischemic stroke and constitutes a fundamental aspect in the physiology and pathology of this condition (Jayaraj et al., 2019). Inflammation relies on various cell types, including microglia, astrocytes, cerebrovascular endothelial cells, mast cells, and leukocytes, to reach the cerebrospinal fluid through the abnormally permeable blood-brain barrier (Das Sarma, 2014). It is correlated with the initiation of inflammatory signaling pathways and the secretion of proinflammatory cytokines (Figure 1).

Nuclear factor kappa B (NF-κB) plays a crucial role in the modulation of immune response and inflammatory processes (Sun, 2017). Under normal conditions, The cytoplasmic IkB protein binds to NF-κB and facilitates its translocation into the nucleus in response to proinflammatory stimulation (Singh and Singh, 2020), thereby governing the regulation of target gene expression. Inhibition of NF-κB reduces inflammatory responses in a mouse model (Ho et al., 2023), the induction of NF-κB signaling mitigates the inflammatory response associated with cerebral ischemia-reperfusion injury in an experimental rat model (Li J et al., 2024), and treatment with NF-κB p65 inhibitors after ischemic stroke partially reverses the upregulation of proinflammatory cytokines (Qian Y et al., 2024). The findings imply that targeting NF-κB could be a viable therapeutic approach for managing ischemic stroke. The interplay between innate immunity regulation and energy metabolism is mediated by the reciprocal crosstalk of NF-κB and Sirt1 signaling pathways, with NF-κB pathway being pivotal in mounting defense against innate immune threats while Sirt1 governs cellular survival and oxidative respiration (Kauppinen et al., 2013). Ischemic stroke is characterized by the interaction between oxidative stress and inflammation, forming a detrimental cycle of oxidative stress-inflammation in its pathological mechanism. The activation of glycolytic energy by NF-κB occurs during inflammation, while Sirt1 governs the regulation of inflammation and oxidative stress. The activation of Sirt1 suppresses NF-κB signaling and promotes oxidative metabolism and resolution of inflammation. The inhibition of Sirt1 can be attributed to the disruption of oxidative energy metabolism and stimulation of NF-κB-induced inflammatory responses, which are commonly observed in various chronic metabolic and age-related diseases. Surprisingly, the activation of adenosine monophosphate (AMP)-activated protein kinase (AMPK), peroxisome proliferator-activated receptor gamma coactivator (PGC-1α), and peroxisome proliferator-activated receptor α (PPARα) has been found to suppress NF-κB signaling by Sirt1. The expression and signal transduction of Sirt1 are downregulated by NF-κB in response to oxidative stress, mediated through reactive oxygen species (ROS), interferon-γ (IFN-γ), and poly (ADP-ribose) polymerase 1 (PARP-1) (Kauppinen et al., 2013). The RelA/p65 subunit of NF-κB is targeted by the Sirt1 protein, leading to deacetylation of RelA/p65 (Yeung et al., 2004) and subsequent inhibition of NF-κB activity. The acetylation also inhibits the methylation of neighboring lysine residues (K314 and K315), thereby facilitating the ubiquitination and subsequent degradation of p65 (Yeung et al., 2004). In addition, the phosphorylation of its transcription activator can be inhibited by Sirt1, leading to the inhibition of NF-κB. The inhibition of NF-κB by Sirt1 may potentially mitigate the progression of neuroinflammation in ischemic stroke.

The high mobility group protein B1 (HMGB1) is actively secreted by activated immune cells or passively released into the extracellular environment by dead or injured cells, and it has the ability to interact with a diverse range of target cell receptors. Cerebral ischemic injury can induce the reactivity of microglia cells and actively release acetylated HMGB1, leading to glial-neuron neuroinflammation (Wei L et al., 2022). Sirt1 has been studied in different fields to regulate the hyperacetylation of HMGB1 and inhibit the translocation of HMGB1. The release and nuclear translocation of HMGB1 are intricately linked to the deacetylase activity of Sirt1, which has identified HMGB1 as a novel target for deacetylation by Sirt1. The regulation of HMGB1 acetylation, mediated by Sirt1, constitutes a crucial step in the lipopolysaccharide (LPS)-induced release of HMGB1. The acetylation of HMGB1 induced by LPS or p300/CBP associated factor (PCAF) can be effectively targeted by Sirt1, leading to the inhibition of HMGB1 release in macrophages through its deacetylation process. The acetylation of HMGB1 has been demonstrated to be elevated in mouse embryonic fibroblasts derived from Sirt1 knockout mice, and the overexpression of Sirt1 completely counteracts this increase (Hwang et al., 2014). The upregulation of Sirt1 regulates the acetylation status of HMGB1, which in turn plays a crucial role in the cellular response to inflammation by controlling the release of HMGB1 through deacetylation. The physical interaction between HMGB1 and Sirt1 can lead to the formation of a complex. The lysine residues of HMGB1 play a crucial role in its interaction with Sirt1, and the acetylation of HMGB1 on these lysine residues mediated by inflammatory signaling promotes the relocation of HMGB1 to the cytoplasm. The nuclear proteins are transformed into cytokines in response to inflammatory stimuli. The protein Sirt1 physically interacts with multiple lysine residues within the nuclear localization signal (NLS) site of HMGB1 through its N-terminal lysine residues, leading to the deacetylation of HMGB1. This process results in the retention of HMGB1 within the nucleus and a reduction in its translocation to the cytoplasm, thereby inhibiting the release of HMGB1 and subsequently improving inflammation (Xu et al., 2014; Zainal et al., 2017). Studies have shown that melatonin reduces HMGB1 production through Sirt1 signaling activation, alleviates brain edema and neuronal apoptosis, and maintains the integrity of the blood-brain barrier (Zhao et al., 2015). The Sirt1-dependent regulation of HMGB1 in hypoxia-reperfusion-injured brain microvascular endothelial cells contributes to the amyloid-producing pathway in neurons (Lee et al., 2020). By enhancing the activity of Sirt1, ω-3 PUFA facilitates a direct interaction between Sirt1 and HMGB1. This interaction effectively suppresses the inflammatory response, thereby preventing neuronal apoptosis and promoting neuroprotection (Chen et al., 2018). Hyperbaric oxygen improves cerebral ischemia-reperfusion injury by mediating Sirt1-induced deacetylation of HMGB1 (Zhao P. C. et al., 2021). Hence, targeting the Sirt1/HMGB1 pathway could potentially offer a novel therapeutic approach for addressing neuroinflammation.

The activation of the NLRP3 inflammasome is intricately associated with neuroinflammation and neurodegeneration, thereby exacerbating brain damage following ischemic stroke. The inflammasome is a complex of cytoplasmic molecules. After receiving intracellular signals, the cytoplasmic sensor NLR triggers the formation of the inflammasome complex. Subsequently, activated caspase-1 processes and converts pro-IL-1β and pro-IL-18, which are precursors of interleukins. This process leads to the release of IL-1β and IL-18, which are cytokines associated with inflammation (Rathinam and Fitzgerald, 2016). A growing body of research has demonstrated that Sirt1 exerts its anti-inflammatory effects by inhibiting the activation of NLRP3 inflammasome. Resveratrol (RSV), an activator of Sirt1, reduces inflammatory response and alleviates traumatic brain injury (TBI) by activating Sirt1 to reduce ROS production and inhibit NLRP3 activation (Zou et al., 2018). The protective effect of RSV against cerebral ischemia-reperfusion injury is mediated through the inhibition of NLRP3 inflammasome activation via Sirt1-dependent autophagy (He et al., 2017). The activation of Sirt1 hinders the activation of NLRP3 inflammasome and subsequent cleavage of caspase-1, as well as secretion of IL-1β. However, the activation of NLRP3 inflammasome was significantly increased upon Sirt1 knockdown (Li et al., 2017). The activation of the NLRP3 inflammasome and the conversion of pro-caspase to caspase-1, which in turn converts pro-IL-1β to IL-1β, are directly inhibited by Sirt1 (Li et al., 2017). The application of transcutaneous electrical acupoint stimulation can effectively suppress neuroinflammation by modulating the Sirt1/NLRP3 signaling pathway following ischemic stroke, thereby mitigating cerebral injury (Tan et al., 2024). The activation of NLRP3 inflammasome via Sirt1 is inhibited by safflor extract, leading to a reduction in neuroinflammation in a mouse model of brain injury (Shaheen et al., 2021). Therefore, the Sirt1/NLRP3 signaling pathway may attenuate the development of neuroinflammation in ischemic stroke.

### 2.2 Sirt1 and oxidative stress

**FIGURE 2:**
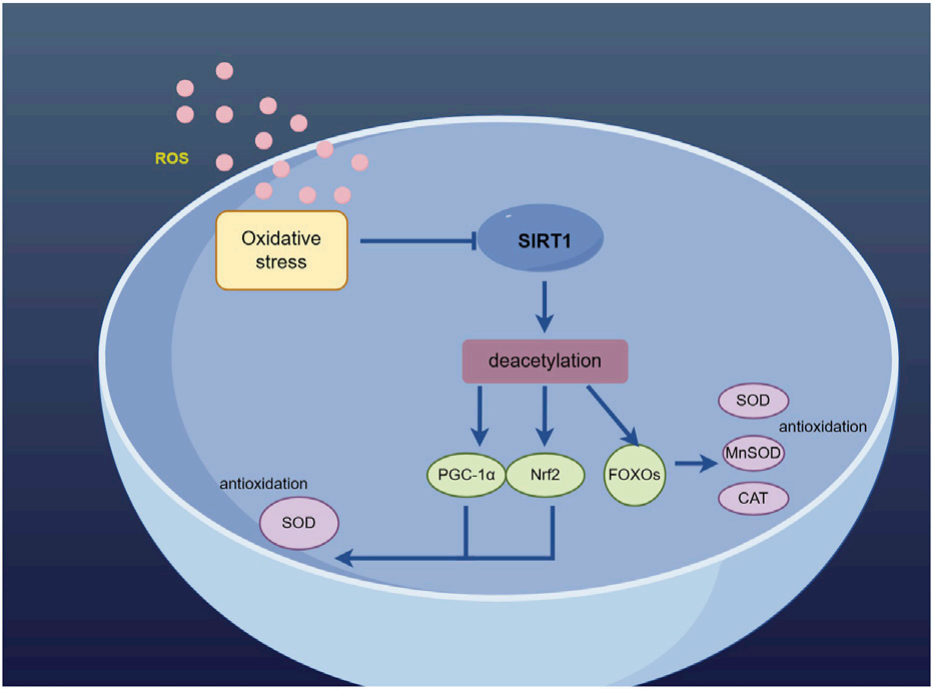
Sirt1 deacetylates PGC-1α, Nrf2 and FOXO to activate the antioxidant defense mechanism. (By Figdraw).

The process of ischemic stroke initiates oxidative stress, which triggers a series of cellular and molecular events leading to neurodegeneration and neuronal death. Compared to other bodily organs, neurons in the brain are more susceptible to reactive oxygen species (ROS) due to their relatively limited presence of endogenous antioxidants (Coyle and Puttfarcken, 1993), and the increase of ROS in cells can lead to oxidative stress and mitochondrial dysfunction, which further aggravates brain damage (Mattiasson et al., 2003). After cerebral ischemia-reperfusion, oxidative stress may lead to parenchymal hemorrhage, vasogenic brain edema, etc. Therefore, it is crucial to find the targets that regulate oxidative stress in ischemic stroke (Figure 2).

PGC-1α is a critical factor in maintaining neuronal survival and synaptic transmission. It acts as a coactivator for peroxisome proliferator-activated receptor γ, thereby mitigating oxidative stress damage through scavenging excessive ROS, inducing the expression of antioxidant enzymes, and preserving mitochondrial function. The expression of PGC-1α is particularly high in tissues characterized by active oxidative metabolism, notably in the brain (Lin et al., 2005). An increasing number of research findings have indicated the impact of Sirt1 on PGC-1α expression, and the introduction of Sirt1 siRNA transfection not only reduces the levels of Sirt1 but also affects PGC-1α expression (Yue et al., 2016). So far, Sirt1 is the sole protein with the ability to bind to PGC-1α and remove acetyl groups from it both in living organisms and in laboratory settings. Sirt1 interacts specifically with the region of PGC-1α encompassing amino acid residues 200–400, leading to deacetylation of PGC-1α through a process that relies on NAD. This interaction ultimately enhances the activation of PGC-1α. Furthermore, utilize it as a deacetylation substrate (Huang et al., 2014). The activation of Sirt1/PGC-1α signaling pathway leads to the activation of antioxidant defense mechanisms, as PGC-1α acts as a downstream effector of Sirt1 and exerts neuroprotective effects. The efficacy of RSV in the treatment of ischemic stroke has been demonstrated through its ability to enhance the Sirt1/PGC-1α signaling pathway. The expression of Sirt1/PGC-1α can be enhanced by Icariin, thereby exerting a preventive effect on ischemic stroke (Zhu et al., 2010). When Sirt1 is activated, it can deacetylate downstream PGC-1α, thereby exerting an antioxidant role as one of the targets of Sirt1. The induction of ROS detoxification enzymes by PGC-1α, one of the targets of Sirt1, enables neurons to effectively mitigate oxidative stress through the increase in Sirt1-mediated PGC-1α levels (St-Pierre et al., 2006).

Nrf2, a vital controller of intrinsic antioxidant defenses, plays a pivotal role in preserving cellular REDOX balance and ensuring homeostasis (Liu et al., 2019). Under oxidative stress conditions, The translocation of Nrf2 into the nucleus triggers the activation of Nrf2-reactive antioxidant genes, leading to the production of protective agents against oxidative damage (Cho et al., 2006). The literature suggests that Nrf2 may function as a downstream effector of Sirt1 signaling, collaborating with Sirt1 to regulate levels of oxidative stress (Huang et al., 2013). On one hand, Sirt1 can enhance the transcription of Nrf2, thereby resulting in an elevation of protein levels. Conversely, Sirt1 has the ability to diminish Nrf2 polyubiquitination by reducing Keap1/Cul3 expression and enhancing Nrf2’s affinity for antioxidant response elements (AREs) (Chung et al., 2021). The regulation of the Sirt1/Nrf2 signaling pathway by sizing exerts a neuroprotective effect in cerebral ischemia-reperfusion through the inhibition of oxidative stress (Mei et al., 2022). Isoglycyrrhizin increases the activation of Nrf2 by activating Sirt1 to reduce oxidative damage induced by subarachnoid hemorrhage (Liu J Q et al., 2022). Studies conducted on animals have demonstrated that the activation of Sirt1 has the potential to impact cellular oxidative state by stimulating the Nrf2/Keap1 pathway, leading to an increase in both total and nuclear levels of Nrf2. This, in turn, enhances the transcriptional activity of Nrf2 (Wang et al., 2023), thereby playing a crucial role in regulating oxidative stress. Based on the aforementioned research, it is suggested that targeting the Sirt1/Nrf2 signaling pathway could be a promising approach to regulate oxidative stress in cases of ischemic stroke.

Class O forkhead transcription factors (FOXOs) have been demonstrated to be essential in the control of oxidative stress, facilitating the management of oxidative stress and promoting resistance against oxidative stress (Hwang et al., 2013), The transcriptional activity of FOXO is commonly controlled through its post-translational modifications, including phosphorylation and acetylation. The attenuation of oxidative stress is mediated by Sirt1 through the regulation of nuclear shuttling and FoxO transcriptional activity. The regulation of FOXO transcription factors by Sirt1 can enhance the promotion of autophagy for biomacromolecules damaged by oxidation. Under oxidative stress, FoxO translocalizes to the nucleus to interact with Sirt1. Sirt1 has the ability to suppress FoxO function and shield cells against oxidative stress (Hou et al., 2011). The regulation of FOXO function is mediated by NAD-dependent deacetylation in response to oxidative stress. Sirt1 enhances cellular resilience against oxidative stress through the deacetylation and stimulation of FOXO, thereby reducing susceptibility to oxidative damage (Brunet et al., 2004). Prior research has established that mammalian Sirt1 plays a vital role in the functioning of FOXO under conditions of oxidative stress by means of NAD-dependent deacetylation, thereby potentially enhancing cellular resilience to stress and promoting longevity (Hisahara et al., 2005). The deacetylation of FOXO3 by Sirt1 can effectively inhibit FOXO3-induced cell death, enabling cells to withstand oxidative stress and induce cell cycle arrest (Fang et al., 2022). SRT1720 functions as a Sirt1 activator to mitigate oxidative stress induced by D-galactose through the reduction of ROS generation via the upregulation of Sirt1 and FOXO3a expression. Additionally, cellular protection against this damage is provided by Sirt1(Yin et al., 2022). In addition, studies have demonstrated that the deacetylation of FoxO1 by Sirt1 exhibits a neuroprotective impact on oxidative stress following cerebral ischemia-reperfusion (Yan et al., 2019). Sirt1 serves as an upstream mediator in the signal transduction pathway of FoxO1, exerting its influence by deacetylating FoxO1 and modulating its DNA binding affinity and promoter binding specificity. Consequently, this process further enhances cellular resistance to oxidative damage (Lin et al., 2022). Surprisingly, the Sirt1 promoter region has been found to be a direct binding site for FoxO1. This interaction leads to the regulation of Sirt1transcription by FoxO1 and subsequently results in an elevation of Sirt1 expression (Xiong et al., 2011).

### 2.3 Sirt1 and autophagy

**FIGURE 3:**
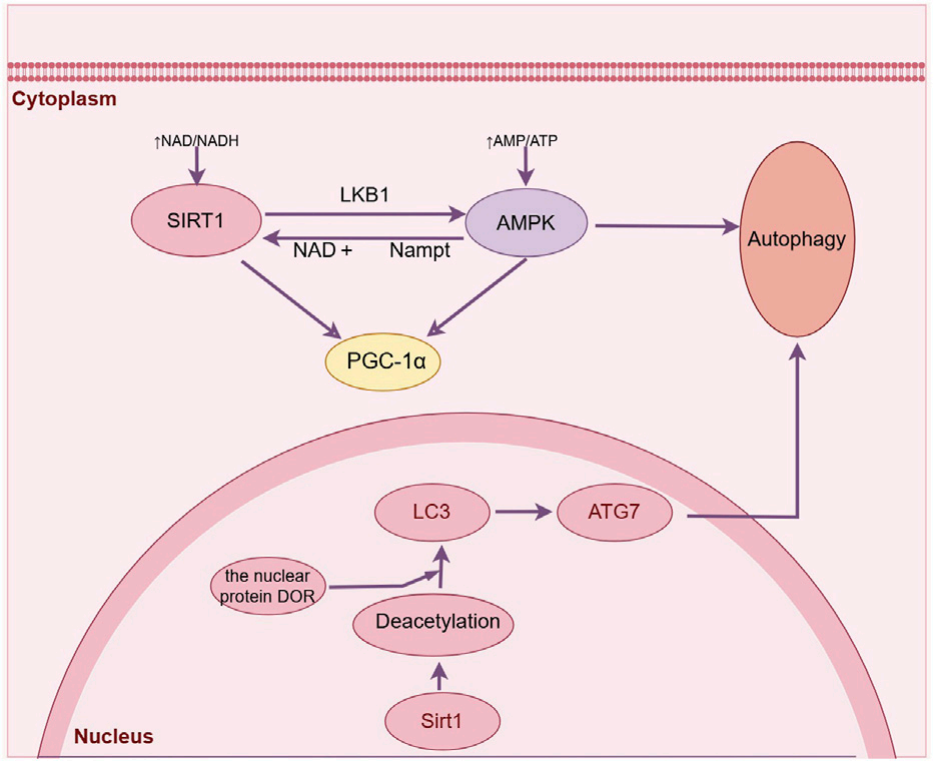
Sirt1 can directly regulate the deacetylation of autophagy related proteins and induce autophagy after ischemic stroke. (By Figdraw).

Autophagy is a mechanism involving lysosomes that facilitates the breakdown and reutilization of cellular components, specifically targeting those that have undergone misfolding or aggregation upon initiation (He and Klionsky, 2009). The process of autophagy plays a pivotal role in maintaining neuronal health and protein homeostasis within the mammalian nervous system, particularly in neurons, by effectively eliminating large, insoluble protein aggregates (Bento et al., 2016). Currently, despite the recognition of autophagy as a pivotal mechanism in ischemic stroke (Mo et al., 2020), The precise role of autophagy in the pathogenesis of ischemic stroke still requires further elucidation and remains consistent. The consensus is that moderate activation of autophagy confers neuroprotective effects, whereas excessive activation of autophagy proves detrimental. The activation of autophagy in the early stage of ischemic stroke serves as a self-protective mechanism, exerting a neuroprotective effect through the degradation of misfolded proteins and damaged organelles to maintain intracellular homeostasis (Mo et al., 2020). However, suppose the brain reperfusion defect is not corrected. In such circumstances, the persistence of a heightened state of “autophagic stress” will result in the stimulation of numerous lysosomes and ultimately culminate in cellular demise (Chu, 2006) (Figure 3).

Acetylation serves as a crucial regulatory process for autophagy, governing the initiation of autophagy and the formation of autophagosomes (Xu and Wan, 2023). The activity of Sirt1 impacts the extent of protein acetylation, and this post-translational modification is intricately linked to the process of autophagy. Sirt1 enhances autophagy by removing acetyl groups from autophagy-related proteins including ATG5, ATG6, ATG7, and LC3 during periods of nutrient deprivation. LC3 plays a vital role in initiating autophagy by shuttling back and forth between the nucleus and cytoplasm, while LC3-II/LC3-I serves as an essential molecular indicator for assessing autophagic activity (Atya et al., 2024). Its activation in the nucleus is specifically triggered by Sirt1 deacetylation under conditions of starvation, facilitating the interaction between LC3 and the nuclear protein DOR. Consequently, LC3 can relocate to the cytoplasm, enabling its involvement in initiating autophagy (Huang et al., 2015). The findings of various studies have demonstrated that Sirt1 has the ability to augment macroautophagy in astrocytes through the upregulation of LC3 expression, thereby leading to an enhancement in functional recovery following brain injury (Zhang et al., 2019), and melittin can exert neuroprotective effects through Sirt1 activation-mediated deacetylation of LC3 and BECN1 proteins (Martínez-Chacón et al., 2023). The silencing of Sirt1 through siRNA transfection and inhibition using the Sirt1 inhibitor EX527 resulted in a significant reduction in the expression level of LC3II/I, indicating that both SIRT1 silencing and inhibition can effectively attenuate propofol-induced autophagy (Martínez-Chacón et al., 2023). Additionally, it has been reported that Sirt1 effectively enhances the activity of chaperon-mediated autophagy, a third type of autophagy crucial in nerve injury, through its regulation of the deacetylation and ubiquitination processes involving the molecular chaperone DnaJ heat shock protein family member B1 (Dnajb1). Contributes to neuroprotection after closed craniocerebral injury (Zhang Z et al., 2022). The direct regulation of autophagy-related protein deacetylation and interaction with essential components of the autophagy machinery enable Sirt1 to exert precise control over the process of autophagy.

AMPK is a protein kinase that exhibits resistance against cerebral ischemic injury by activating catabolic pathways and inhibiting ATP-utilizing processes (Jiang et al., 2018). Its pre-activation has demonstrated the ability to enhance neural autophagy and mitigate damage caused by ischemic injury (Manwani and McCullough, 2013). An increasing number of studies have provided evidence supporting the interplay between Sirt1 and AMPK, showcasing their significant association in governing autophagy regulation through diverse mechanisms. The liver kinase B1 (LKB1) serves as a key upstream regulator of AMPK, with Sirt1 facilitating the deacetylation and subsequent activation of LKB1. Consequently, the phosphorylation of AMPK is augmented, thereby resulting in an upregulation of AMPK activity downstream and consequently enhancing the neuroprotective effect mediated by AMPK (Lan et al., 2008). Furthermore, the cellular activation of AMPK results in an elevation of the NAD+/NADH ratio, thereby triggering the activation of Sirt1 and facilitating the deacetylation process on various targets like PGC1α. This subsequently enhances the overall activity level of Sirt1 (Cantó et al., 2009). The activation of AMPK can also be induced by Sirt1 as a downstream molecule, thereby promoting autophagy through the activation of AMPK. Melatonin treatment increases Sirt1 activity and AMPK levels, thereby enhancing nerve cells’ autophagic activity and promoting motor function recovery after SCI (Gao K et al., 2020). RSV, functioning as a Sirt1 activator, mitigates cerebral edema and neurobehavioral disorders via the Sirt1/AMPK-mediated autophagy signaling pathway (Li and Han, 2018). By activating Sirt1, RSV increased AMPK phosphorylation in the rat cerebral cortex, reduced infarct size, and promoted mitochondrial autophagy in neuron cultures (Pineda-Ramírez et al., 2020). Thus, Sirt1/AMPK-mediated signaling may provide a potentially useful strategy to modulate autophagy during ischemic stroke.

Notably, Sirt1 induces autophagy following ischemic stroke and exerts a negative regulatory impact on this cellular process. Betulinic acid activates the Sirt1/FOXO1 pathway while inhibiting autophagy, thereby ameliorating brain damage subsequent to ischemic stroke (Zhao Y et al., 2021). The natural compound magnoflorine, possessing antioxidant and immunomodulatory properties, has been discovered to exert a preventive effect against ischemic stroke by activating the Sirt1/AMPK pathway in order to inhibit autophagy (Liang et al., 2022). Hence, the presence of Sirt1 is crucial for maintaining autophagy levels during ischemic stroke.

### 2.4 Sirt1 and mitochondrial dysfunction

**FIGURE 4:**
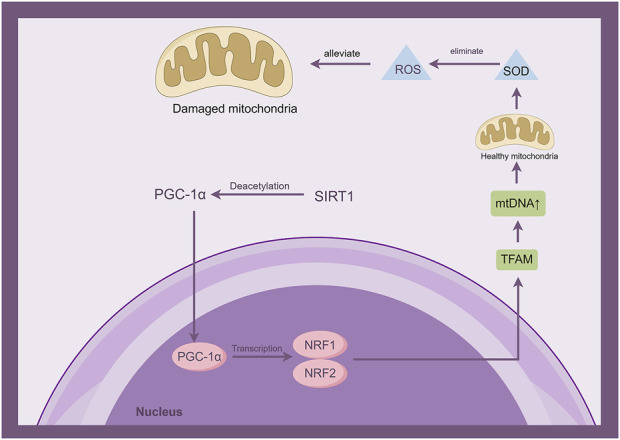
Sirt1 is involved in the development of mitochondrial dysfunction by activating the interaction of PGC-1α with mitochondrial transcription factor A (TFAM) through deacetylation. (By Figdraw).

The imbalance between mitochondrial fission and fusion following an ischemic stroke may disrupt the homeostasis of mitochondria, leading to mitochondrial dysfunction (Li et al., 2022). The occurrence of mitochondrial damage following ischemic injury can result in diminished energy production, excessive accumulation of free radicals, and the onset of oxidative stress, ultimately culminating in neuronal death. Mitochondrial impairment is a prominent characteristic of cerebral ischemia/reperfusion injury and the resulting neuronal demise. Consequently, addressing mitochondrial dysfunction could be considered as an approach to manage ischemic stroke (Twig and Shirihai, 2011) (Figure 4).

The transcription factors activated by PGC-1, such as PPAR, NRF, and ERR, play a crucial role in the regulation of mitochondrial biogenesis. This process involves the controlled transcription of genes encoded by both mitochondrial and nuclear DNA. The effector of PGC-1 regulates mitochondrial DNA and gene expression, while deacetylation of PGC-1 by Sirt1 enhances its activity. Therefore, Sirt1 functions as a REDOX and metabolic sensor to regulate the process of mitochondrial biogenesis and prevent the occurrence of mitochondrial dysfunction (Qian L et al., 2024). The PGC-1 family comprises three individuals, specifically PGC-1α, PGC-1β, and the coactivator related to PGC. Among these members, PGC-1α is widely acknowledged as the primary controller of mitochondrial generation and removal. PGC-1α and Sirt1 are localized in the cytoplasmic mitochondrial matrix. The expression of PGC-1α is upregulated and its acetylation is reduced through Sirt1-mediated regulation, thereby activating the interaction between PGC-1α and mitochondrial transcription factor A (TFAM). Thus, augmenting the co-activation of TFAM and facilitating more efficient transcription of mitochondrial DNA (mtDNA) (Qian L et al., 2024). Research has indicated that in the model of ischemic stroke, the Notoginseng triterpenes have been found to regulate the Sirt1/PGC-1α pathway, thereby inhibiting mitochondrial damage. This regulation leads to protection of mitochondria and a notable enhancement in neuronal survival rates (Xie et al., 2023). C1q/tumor necrosis factor-related protein-3 (CTRP3) safeguards hippocampal neurons from mitochondrial damage caused by ischemic stroke via the Sirt1/PGC-1α signaling pathway. It also enhances mitochondrial biogenesis and physiological function while regulating any potential dysfunction (Gao J et al., 2020). The administration of Nicotinamide adenine dinucleotide (NAD) promotes the expression of Sirt1/PGC-1α, thereby mitigating mitochondrial damage induced by cerebral hypoxia. In a mouse model of ischemic stroke, the Sirt1/PGC-1α pathway was activated by intranasal delivery of mitochondria, resulting in reduced brain damage. This intervention significantly decreased cerebral infarct volume and alleviated brain edema (Salman et al., 2024). The Sirt1/PGC-1α signaling pathway may serve as a promising therapeutic target for the regulation of mitochondrial dysfunction in ischemic stroke.

### 2.5 Sirt1 and pan-apoptosis

The term PANoptosis refers to a complex form of cell death crosstalk, which is characterized as a programmed cell death pathway (PCD) that incorporates critical features of pyroptosis, apoptosis, and necroptosis. However, none of these PCD pathways can fully account for it (Wang and Kanneganti, 2021). Emerging data indicates that the involvement of these three PCD pathways in the development of ischemic stroke is becoming increasingly evident, and inhibiting them has shown potential in reducing damage to the brain caused by ischemia (Tuo et al., 2022). The findings of a comprehensive study on brain ischemia/reperfusion injury indicate the presence of PANoptosis in cases of ischemic brain injury (Yan et al., 2022), The targeting of PAN apoptosis may potentially mitigate neurological damage following ischemic stroke, thereby enhancing prognosis and facilitating recovery.

#### 2.5.1 Sirt1 and apoptosis

Apoptosis is a process of cell death that is pre-programmed. After ischemic stroke, neurons undergo apoptosis due to oxidative stress, excitotoxicity, and inflammation within days to weeks (Khoshnam et al., 2017). Apoptosis can lead to neuronal apoptosis and further aggravate brain damage. One of the primary roles played by the SIRT protein family is its involvement in the intricate process of apoptosis. The inhibition of Sirt1 can exacerbate cerebral ischemic injury, accompanied by an augmented acetylation of P53 and NF-κB P65, which are pivotal factors in the apoptotic pathway leading to brain injury. hence, the involvement of Sirt1 is crucial in the process of cellular apoptosis during ischemic stroke.

P53, a transcription factor involved in the cellular response to stress, was identified as one of the initial targets for Sirt1-mediated deacetylation (Luo et al., 2001). It plays a crucial role in regulating apoptosis. Sirt1is involved in the modulation of p53 protein by directly removing acetyl groups, leading to deacetylation of p53 at Lys379 and suppression of p53-mediated apoptosis (Vaziri et al., 2001), and p53 expression gradually decreases with the increase of SIRT1 level. In addition, SIRT1 has the ability to modulate the p53 signaling pathway through its interaction with proteins. Moreover, *in vitro* experiments have demonstrated that an increase in SIRT1 expression results in a notable decrease in both mRNA and protein levels of molecules associated with the p53 signaling pathway (Wei X et al., 2022). In mouse experiments, the deacetylation activity of SIRT1 inhibits the p53-induced apoptosis pathway, thereby exerting a neuroprotective effect in the prevention of cerebral ischemic injury (Hernández-Jiménez et al., 2013). Cycloastragalool reduces p53 acetylation in the ischemic brain by upregulating Sirt1, thereby inhibiting cell apoptosis in ischemic stroke (Li M et al., 2020). Activation of Sirt1 by curcumin induces p53 deacetylation, leading to apoptosis in cerebral ischemia/reperfusion injury (Zendedel et al., 2018). After activation of Sirt1, RSV regulates p53-related downstream pro-apoptotic molecules, alleviates brain edema, and protects neurons by improving the blood-brain barrier and p53 deacetylation (Qian et al., 2017). Activation of Sirt1 in brain microvascular endothelial cells by nicotinamide mononucleotide adenylate transferase 1 (NMNAT1) significantly reduces acetylated p53 in ischemic microvessels, Thereby, the occurrence of apoptosis in microvessels during cerebral ischemia is decreased, leading to a reduction in blood-brain barrier damage following an ischemic stroke (Zhang Y et al., 2022). Therefore, Sirt1 may mediate cell apoptosis in ischemic stroke by inhibiting acetylated p53.

miRNA, a subtype of non-coding RNA, can inhibit protein translation in apoptosis (Wu et al., 2023). Sirt1 has been subjected to the influence of miRNAs, including but not limited to miR-34a, miR-181, miR-128, miR-449, and miR-30a-5p. Sirt1 is critical in protecting from miR-34a-5p inhibition during apoptosis (G. Wang et al., 2016). The level of miR-149-5p is significantly decreased at 24 h following cerebral ischemia-reperfusion injury, and its activity can be increased by resveratrol, a natural activator of Sirt1, accompanied by the downregulation of P53 and caspase-3. This means that miR-149-5p regulates caspase-3-mediated neuronal apoptosis through Sirt1 (Teertam et al., 2020). miR-489-3p has been identified as a key player in the regulation of Sirt1-mediated apoptosis in cerebral ischemia cells. Research indicates that upregulation of miR-489-3p exacerbates neuronal apoptosis caused by cerebral ischemia-reperfusion injury through its targeting of Sirt1 (Song et al., 2022). Modulating the signaling pathway of miR-142-3p/Sirt1 can effectively suppress cell necrosis and apoptosis triggered by oxygen-glucose deprivation followed by reoxygenation, thereby enhancing recovery from cerebral ischemia-reperfusion injury (Meng et al., 2023). The promotion of inflammation and apoptosis in hypoxic-ischemic brain injury is facilitated by miR-155 through its targeting of Sirt1(Ke et al., 2023). In addition, an animal experiment found that MiR-146b reduced cell apoptosis in brain tissue by targeting Sirt1 and played a protective role in rats with cerebral infarction (Yang and Zeng, 2023). Targeting MiRNA Sirt1 could be a promising approach to modulate apoptosis in cases of ischemic stroke.

#### 2.5.2 Sirt1 and pyroptosis

Pyroptosis is a mode of cell death that is both programmed and inflammatory, with inflammasome activation playing a crucial role in regulating this process. During ischemic stroke, pyroptosis can directly promote neuronal death, thereby aggravating neuroinflammation and neurodegeneration and aggravating brain tissue damage. Therefore, pyroptosis cannot be ignored in ischemic stroke.

The activation of Caspase-1 and release of proinflammatory cytokines IL-1β and IL-18, leading to pyroptosis, is facilitated by the NLRP3 inflammasome. The expression of proinflammatory cytokines is inhibited by Sirt1, which can inhibit the inflammatory response of NLRP3 by regulating Nrf2. The deacetylation of the transcription factor NF-κB by Sirt1 also serves as an inhibitory mechanism for NLRP-3 inflammasome activation, effectively regulating both the activation of NLRP3 inflammasomes and their downstream inflammatory molecules. In addition, the activation of NLRP3 inflammasome relies on the presence of ROS and byproducts from mitochondrial degradation. Sirt1, through its protective effects on mitochondria and antioxidant properties, can effectively suppress the activation of NLRP3. Numerous research studies have demonstrated the ability of Sirt1 to impede the activation of NLRP3 inflammasome and subsequent pyroptosis. The administration of sevoflurane enhances Sirt1 expression, leading to a reduction in NLRP3-dependent caspase-1/11-GSDMD pathway-mediated pyroptosis within the hippocampus (Chen et al., 2022). In treating ischemic stroke, RNA binding protein RPS3 regulates microglial pyroptosis and neuronal damage through the Sirt1/NLRP3 pathway (Zhou et al., 2023). LPS promotes pyroptosis by targeting Sirt1 to activate the NLRP3 inflammasome (Li H et al., 2020). In rodents, the Sirt1/NLRP3 signaling pathway is modulated by colchicine to suppress pyroptosis (Li H et al., 2024). Activation of Sirt1 and inhibition of NLRP3 inflammasome-mediated pyroptosis contribute to the protective effects of oxymatrine against human umbilical vein endothelial cell injury induced by oxLDL (Jin et al., 2021). According to the above studies, Sirt1 may mediate pyroptosis in ischemic stroke by inhibiting NLRP3.

#### 2.5.3 Sirt1 and necroptosis

Necroptosis, an independent caspase-mediated programmed cell death pathway, has been found to contribute significantly to the adverse consequences observed during the development of ischemic stroke. Necroptosis has been demonstrated to contribute to and worsen the extent of brain tissue harm following cerebral ischemia and reperfusion injury (She et al., 2020). A rising body of research has indicated that Sirt1 exhibits the potential to hinder necroptosis in diverse disease models, encompassing cancer (Carafa et al., 2018), liver fibrosis (Sun et al., 2022), nephropathy (Liu Z et al., 2022), and others. By activating the Sirt1 pathway, unacylated ghrelin prevents the upregulation of necrotizing apoptotic proteins such as RIP1 and RIP3, thereby safeguarding skeletal muscle against compression-induced damage (Ugwu et al., 2017). The Sirt1 pathway is involved in curcumin-induced endoplasmic reticulum stress-mediated necroptosis in hepatic stellate cells, leading to the alleviation of liver fibrosis (Sun et al., 2022). Significantly, the protective impact on cerebral ischemia-reperfusion injury was weakened by the Sirt1 inhibitor EX-527 via modulation of the necroptosis signaling pathway. It mitigated the disruption caused by ischemia to downstream metabolic enzyme activity associated with necroptosis, ultimately leading to a decrease in infarct volume (Nikseresht et al., 2019). The findings imply that the inhibition of Sirt1 offers neuroprotection against ischemic stroke by suppressing necroptosis. However, there is a paucity of studies investigating the relationship between Sirt1 and necroptosis following ischemic stroke, necessitating further research to elucidate their interaction.

A growing body of evidence suggests that Sirt1is responsible for regulating the three PCD pathways involved in PANoptosis. This regulatory mechanism plays a pivotal role in both the development and treatment of ischemic stroke, underscoring the significance of each distinct mode of cell demise. The precise mechanisms that facilitate communication between these three types of programmed cell death still require a comprehensive understanding. In any case, Sirt1 regulation of the PCD pathway may represent a novel approach to modulating PAN apoptosis in ischemic stroke.

## 3 Conclusions and prospects

Ischemic stroke has garnered considerable interest among healthcare practitioners over the past few years and remains a prominent contributor to global mortality rates. Therefore, new treatment methods are urgently needed. Sirt1 has unique enzymatic activity, physiological function, and subcellular localization, enhances synapse formation and activity, plays a neuroprotective role by regulating fate precursor cells, inhibiting p53, and attenuating axonal degeneration, and is involved in various physiological and pathological processes of ischemic stroke. The inhibition of the NF-κB inflammatory response and NLRP3 inflammasome activation can be achieved through the activation of Sirt1. Sirt1 engages in a molecular interaction with the lysine residues of HMGB1 to facilitate the regulation of neuroinflammation. Sirt1 exhibits properties such as antioxidation, anti-inflammation, and genomic stabilization, enabling it to deacetylate FOXO3 and PGC-1α. We increased Nrf2 transcription and phosphorylation of AMPK. The identification of Sirt1 as a potential therapeutic intervention for ischemic stroke is supported by the data we have collected.

Despite these significant findings, numerous questions still require further investigation. Firstly, Sirt1 is a redox-sensitive protein that undergoes covalent modification under inflammatory and oxidative conditions; therefore, the simple pharmacological activation of Sirt1 may prove ineffective. Although numerous natural compounds, including resveratrol, curcumin, and icariin, have been confirmed to exert neuroprotective effects on cerebral ischemia through Sirt1 activation, further research is warranted (Jiao and Gong, 2020). However, resveratrol exhibits certain limitations. As a polyphenolic activator, it has poor water solubility, which significantly affects its bioavailability (Xu et al., 2018). Therefore, this factor must be carefully considered in clinical trials. It has been reported that high-dose resveratrol not only induces Sirt1-independent activation of AMPK but also results in toxic off-target effects (Price et al., 2012). Although synthetic drugs such as rosuvastatin and simvastatin exhibit neuroprotective effects in ischemic stroke by activating Sirt1, they remain in the experimental pharmacological phase. Additionally, the safety, dose optimization, and potential toxicity of these modulators in humans require further investigation. Therefore, the future should focus on improving the selectivity and bioavailability of novel Sirt1 regulators to precisely regulate Sirt1 activity while minimizing side effects. Second, the biological functions of Sirt1 are both complex and extensive. Sirt1 has been linked to increased blood-brain barrier (BBB) permeability and heightened energy consumption. Consequently, the question of whether targeting Sirt1 for the treatment of ischemic stroke by increasing its levels would be beneficial to brain tissue under ischemic stress remains a topic of debate. Recent data suggest that Sirt1 inhibition, rather than its activation, exerts a neuroprotective effect in specific settings. Conflicting results indicate that the subcellular localization of Sirt1 may influence its role in regulating cell death. Therefore, it is imperative to further investigate the role of Sirt1 in ischemic stroke across various brain cell types, elucidate the precise mechanisms of action, and draw robust conclusions. Third, the relationship between Sirt1 genetic polymorphisms and ischemic stroke has not been extensively investigated, with many existing studies focusing on animal models. Future prospective clinical studies should be designed to explore the expression profiles and activity changes of Sirt1 and its related targets in ischemic stroke, and to identify novel diagnostic and prognostic markers. Fourth, while the three programmed cell death (PCD) pathways of PANoptosis have been demonstrated to occur simultaneously in various experimental ischemic stroke models, there is currently no evidence to confirm whether PANoptosis is triggered in every ischemic stroke patient. Although Sirt1 regulates these three PCD pathways, the precise molecular mechanisms and interacting proteins involved in the crosstalk between these pathways remain unclear. Further studies are necessary to elucidate these interactions and confirm their relevance in clinical settings.

In brief, Sirt1 exhibits promising potential as a therapeutic target for ischemic stroke and plays a vital role in reducing the neuropathology associated with this condition, minimizing brain injury, and enhancing stroke prognosis. Further studies are warranted to help develop effective strategies for the treatment of ischemic stroke.
